# MRI, CT and high resolution macro-anatomical images with cryosectioning of a Beagle brain: Creating the base of a multimodal imaging atlas

**DOI:** 10.1371/journal.pone.0213458

**Published:** 2019-03-07

**Authors:** Kálmán Czeibert, Gábor Baksa, András Grimm, Szilvia Anett Nagy, Enikő Kubinyi, Örs Petneházy

**Affiliations:** 1 Department of Ethology, Institute of Biology, Eötvös Loránd University, Budapest, Hungary; 2 Department of Anatomy, Histology and Embryology, Semmelweis University of Medical Sciences, Budapest, Hungary; 3 Department of Otorhinolaryngology and Head and Neck Surgery, Semmelweis University of Medical Sciences, Budapest, Hungary; 4 MTA-PTE Clinical Neuroscience MR Research Group, Pécs, Hungary; 5 Neurobiology of Stress Research Group, Szentágothai Research Center, University of Pécs, Pécs, Hungary; 6 Department of Neurosurgery, Medical School, University of Pécs, Pécs, Hungary; 7 Pécs Diagnostic Centre, Pécs, Hungary; 8 University of Kaposvár, Kaposvár, Hungary; Medical University of Vienna, Austria, AUSTRIA

## Abstract

Most common methods that directly show macro- or microscopic anatomy of the brain usually require the removal of the organ from the neurocranium. However, the brain can be revealed *in situ* by using proper sectioning techniques. Our aim was to both improve the cryosectioning method, test its limits and create a high-resolution macro-anatomical image series of a Beagle brain, which at the time of the study did not exist. A two-year-old female Beagle has been scanned with CT and MRI ante and post mortem, then the arteries of the head were filled with red resin. After freezing to -80°C, a neurocranium block was created and was embedded into a water-gelatin mix. Using a special milling device and a DSLR camera, 1112 consecutive RGB-color cryosections were made with a 100 μm layer thickness and captured in high resolution (300 dpi, 24-bit color, and pixel size was 19.5 x 19.5 μm). Image post-processing was done with Adobe Photoshop CS3 and Thermo Scientific Amira 6.0 softwares, and as a result of the proper alignment and coregistration, visualization and comparing was possible with all the applied imaging modalities (CT, MRI, cryosectioning) in any arbitrary plane. Surface models from the arteries, veins, brain and skull were also generated after segmentation in the same coordinate system, giving a unique opportunity for comparing the two-dimensional and three-dimensional anatomy. This is the first study which focuses directly to this high-definition multimodal visualization of the canine brain, and it provides the most accurate results compared to previous cryosectioning studies, as using an improved method, higher image quality, more detailed image, proper color fidelity and lower artefact formation were achieved. Based on the methodology we described, it can serve as a base for future multimodal (CT, MR, augmented- or virtual reality) imaging atlases for medical, educational and scientific purposes.

## Introduction

Creating a novel anatomical atlas requires a technique showing the organ in a different perspective (e.g. detailedness, staining, and tissue-fidelity), or that the applied method results in enhanced image quality compared to a previous atlas. In order to validate the need for cryosectioning of a canine brain to create a new comparative image series, we provide an overview of the main techniques that make possible considering the requirements mentioned above. There are several ways to visualize macro- or microanatomical structures in anatomy: conventional preparations and sections can be made shortly post mortem on a fresh cadaver, or previously fixed with a fixative agent [1], creating macerated bones and skeletons [2–4], or corrosion casting [5–7]. The result of the tissue preparation can be captured in photographs or videos, or these procedures can be combined with the different imaging methods (like CT or MRI), so that the selected region or specimen can be digitized for 2- and 3-dimensional analysis. These techniques are all suitable methods to visualize the different systems of the body, and they can be grouped by direct/indirect methods and tissue maintaining/tissue destructive methods. Direct imaging means that the original tissue can be seen either in its real color (e.g. with endoscopy) or dyed using histological staining [8]. Indirect imaging, such as computed tomography (CT), magnetic resonance imaging (MRI), positron emission tomography (PET) and single-photon emission computed tomography (SPECT) provides a computer-generated picture (most commonly a grayscale image), and during post-processing an artificial color is added for differentiation based on tissue properties. Examples include X-ray attenuation (CT), or spin and precessing of the proton and water diffusion (MR) [9]. Indirect imaging methods are tissue maintainers, but have some limitations to show the real environment (mostly due to small spatial resolution). The central nervous system is enclosed into a bony capsule, and in the case of larger animals and humans the head cannot be sectioned together with the skull due to the hardness of the bone—although the present methods make it possible to produce histological sections as large as a complete human brain [10,11]. Plastination of the slices is a good method for tissue preservation and selective staining is possible [12,13], but the specimen’s color is partly altered during the procedure and shrinkage could occur [14–16].

If one wants to study the brain *in situ*, then different imaging methods or sectioning of the entire head are required. Diagnostic imaging techniques create post-processed indirect grayscale images and the quality of the CT and MR imaging depends on spatial resolution, signal to noise ratio (SNR) and various artifacts [17–20]. In order to create true-color macro-anatomical sections, which involve the entire neurocranium, there are two main possibilities: slicing the object into layers with a saw/macrotome blade, or to mill the selected volume stepwise and record the resulting surfaces with a camera. In the first case the slices could be handled individually, and their average size (thickness) could vary from centimetres to millimetres. These slices can be preserved through fixation, staining and/or plastination. In contrast, milling removes a layer from the volume’s surface (tissue destructive method), and the consecutive photographs always record the upcoming polished surface. With this procedure layer thickness depends only on the applied milling technique and its precision, ranging from millimetres to micrometres. This method is called cryosectioning, or cryomacrotomisation [21–23]. There have been several human studies in the cryosectioning field [24–26], and an initiative by the National Library of Medicine in 1996, the Visible Human Project (carried out in association with the University of Colorado Center for Human Simulation), used the cryomacrotomisation to visualize an entire male human body [21]. During the past decades similar projects were made in China (Chinese Visible Human, Virtual Chinese Human projects) [27,28], in South-Korea (Visible Korean Human) [22,29], and by others who also used this technique [10]. Cryomacrotomisation of smaller animals, such as mice and rats has also been performed [30,31].

Cryomacrotomisation focuses on showing the macro-anatomical structures of the body, however, mapping the brain on an ultrastructural level and showing its functional connectivity requires other approaches. Below an overview is given regarding integrative projects that develop detailed brain maps on the cellular level and thus complete the macro-anatomical atlases. Several projects study the central nervous system through detailed micro-anatomical reconstructions and computer simulations. The main projects are: (a) the Blue Brain Project (https://bluebrain.epfl.ch); (b) the Human Brain Project (https://www.humanbrainproject.eu); (c) the Human Connectome Project (http://www.humanconnectomeproject.org); (d) the SpiNNaker (http://apt.cs.manchester.ac.uk/projects/SpiNNaker); (e) the BRAIN Initiative (http://www.braininitiative.org); (f) the Brain/MINDS project (https://brainminds.jp/en); (g) and the China Brain Project [32]. Some of these initiatives created structural brain maps with extremely high resolution, e.g. the BigBrain project produced a 3-dimensional reconstruction of the human brain from 7404 histological sections with 20 μm slice thickness, enabling the visibility of cells (in plane resolution of 10 μm, 2400 dpi and 16-bit color depth) [11]. This atlas also comprised the MRI dataset of the same brain. Another project, in the framework of the Allen Institute for Brain Science, created a publicly available multimodal gene expression atlas [33,34]. They presented MRI and DWI images together with 1356 high resolution (1 μm per pixel) histological sections marking 862 individual structures (including both macroscopic structures like gyri and sulci and also making cytoarchitectural parcellations connected to Brodmann areas). Recent technologies, like optical coherence tomography (OCT) or light sheet fluorescence microscopy (LSFM) are capable of showing the biological tissues without destroying them. Tissue maps produced by these technologies serve as good diagnostic tools, for example the OCT is used in ophthalmology [35], cardiology [36] and brain research [37], whilst LSFM visualizes tissues with subcellular resolution [38].

The cryosectioning of an entire dog was first performed in 1999 [39,40], then a study on a whole body of a one-year-old female Beagle in 2014 [23], and a one-year-old short-hair cat in 2018 [41] have been published. We performed our study in order to further develop the cryosectioning technique and to provide the base for a high resolution multimodal comparative canine brain atlas for research, aid graduate and postgraduate trainings (e.g. not only with comparative images, but also with three-dimensional models) and to provide an aid in neurosurgical intervention planning, and to show what kind of major improvements can be achieved compared to the previous studies. First we made *in*- and *ex vivo* CT and MR imaging on a brain of a two-year-old female Beagle dog with adequate T1- and T2-weighted sequences. To be able to contrast the imaging techniques 1112 consecutive, high resolution, full-color images were created from the same animal’s brain in its original position within the neurocranium including the orbits during cryosectioning. This made possible the precise comparison and analysis of the individual structures in the identical coordinate system.

## Materials and methods

### Subject

In order to ensure that our results were comparable with other studies that show normal anatomical variations [23,42], and to be in accordance with previous widely accepted laboratory studies, we used the Beagle breed as a basic model animal for our study in accordance with the 3R-principle [43]. The subject, a healthy, two-year-old female Beagle dog, weighted 13.5 kg, and was vaccinated and treated against parasites according to the standard veterinary program. The research was performed in accordance with the international recommendations [44]. The animal was obtained from an official research company (National Research Institute for Radiobiology and Radiohygiene, Department of Radiobiology, Division of Animal Experiments and Experimental Animal House), which had the right to keep laboratory animals according to EU regulations and welfare criteria, in a standard kennel environment. The diagnostic imaging and euthanasia were performed on the same day upon receiving the animal, so no separate housing was necessary. The *in vivo* neuroimaging procedures were carried out under general anesthesia and all efforts were made to minimize discomfort for the animal. All husbandry and experimental procedures were approved by the Institutional Ethics Committee and the Hungarian Directorate for Food Chain Safety and Animal Health (PEI/001/956-4/2013).

### Imaging protocol

The MR imaging was obtained using a 3T Magnetom TIM Trio whole-body MRI scanner (Siemens AG, Erlangen, Germany) with a 12-channel phased array head coil. Under the same anesthetic episode and immediately following the MRI, CT scans were obtained using a Siemens Somatom Perspective 128 slices CT (Siemens AG, Berlin and München 2013). The animal was placed in dorsal recumbency during the MR and CT procedures. Transverse slices (which in human terminology are used as axial slices) were oriented perpendicular to the defined axis of the brain (which was set through the rostral and caudal commissures). In order to avoid any unintentional movements during general anesthesia, and to ensure that both the MR and CT examination took place with the animal in the same position (both ante and post mortem), the dog was placed into a double plastic tube, which fixed the position of the body and the head separately. A system was created using plastic screws to hold the head fixed at the zygomatic arch on both sides and at the nuchal region. The holding device was checked both for radiopacity and MR compatibility to avoid any artefacts during scanning. Anesthesia premedication was performed using 6 ml of Propofol intravenously (Fresenius Kabi Deutschland, Propofol 1% MCT/LCT Fresenius emulsion for injection, 10 mg/ml), via the cephalic vein. After intubation inhalational anesthesia with 2.5 volume concentration (%V/V) isoflurane (Isoflurane USP, Abbots) was performed.

First the MR scanning was accomplished, during which the following sequences were obtained: T2-weighted sequence in sagittal plane (TR = 6000 ms, TE = 90 ms, slice thickness = 2 mm, FOV = 96x160 mm^2^ and a 192x320 matrix with voxel size of 0.5x0.5x2 mm), T2-weighted imaging in transverse plane (TR = 10342 ms, TE = 90 ms, slice thickness = 2 mm, FOV = 96x160 mm^2^ and a 192x320 matrix with voxel size of 0.5x0.5x2 mm), 3D T2-SPACE sequence in transverse plane (TR = 1000 ms, TE = 89 ms, slice thickness = 0.5 mm, FOV = 160x160 mm^2^ and a 324x320 matrix with voxel size of 0.5x0.5x0.5 mm), T1-weighted imaging in transverse plane (TR = 300 ms, TE = 2.8 ms, slice thickness = 3 mm, FOV = 160x160 mm^2^ and a 320x320 matrix with voxel size of 0.5x0.5x3 mm). After the native scans, dynamic contrast-enhanced MR angiography was also performed by giving 3 ml gadobutrol (Gadovist, Bayer Schering Pharma AG, Berlin, Germany), followed by 10 ml saline (0.9% NaCl) intravenously through the cephalic vein. During the angiographic examination a coronal T1-weighted sequence was obtained using the following imaging parameters: TR = 2.9 ms, TE = 1.1 ms, slice thickness = 1 mm, FOV = 270x360 mm^2^ and a 288x521 matrix with voxel size of 0.93x0.7x1 mm). Following the MR imaging the animal was transferred in the same position to the CT unit, then CT scanning was carried out (130 kV, 30 mAs, slice thickness = 1 mm, pitch = 0.5, spiral scanning mode). The head and the cervical region up to the third cervical vertebra were scanned. For reconstruction, a soft tissue specific (J30s kernel) reconstruction and a bone specific (H70s kernel) with voxel size 1x1x1 mm was used.

After the *in vivo* imaging, the animal was euthanized while still under general anesthesia by administering a 1.5 ml mixture (T-61 injection) intravenously (containing 300 mg embutramide, 75 mg mebezonium iodide, and 7.5 mg tetracaine hydrochloride). Two hours later—while the animal was still in dorsal recumbency–both common carotid arteries and external jugular veins were exposed minimally-invasively near to the thoracic inlet, and cannulas were placed into the vessels. The amount of 18–18 ml red colored polyurethane resin (VytaTlex-10, urethane rubber) was injected through the common carotids in order to fill the arterial system of the head. Two and a half hours post mortem the same MR protocol (except MR angiography) was performed on the cadaver to provide the possibility for ante and post mortem comparison and to check the brain for the resin-filling. Following the repeated MR scanning the dog was placed into a -80°C deep freezer (without applying cryo-protective agents, based on studies with the same decision [23,39]), to minimize further lytic process taking place after death, and to prepare the tissues for the cryosectioning.

### Blocking and embedding

**Fig 1:**
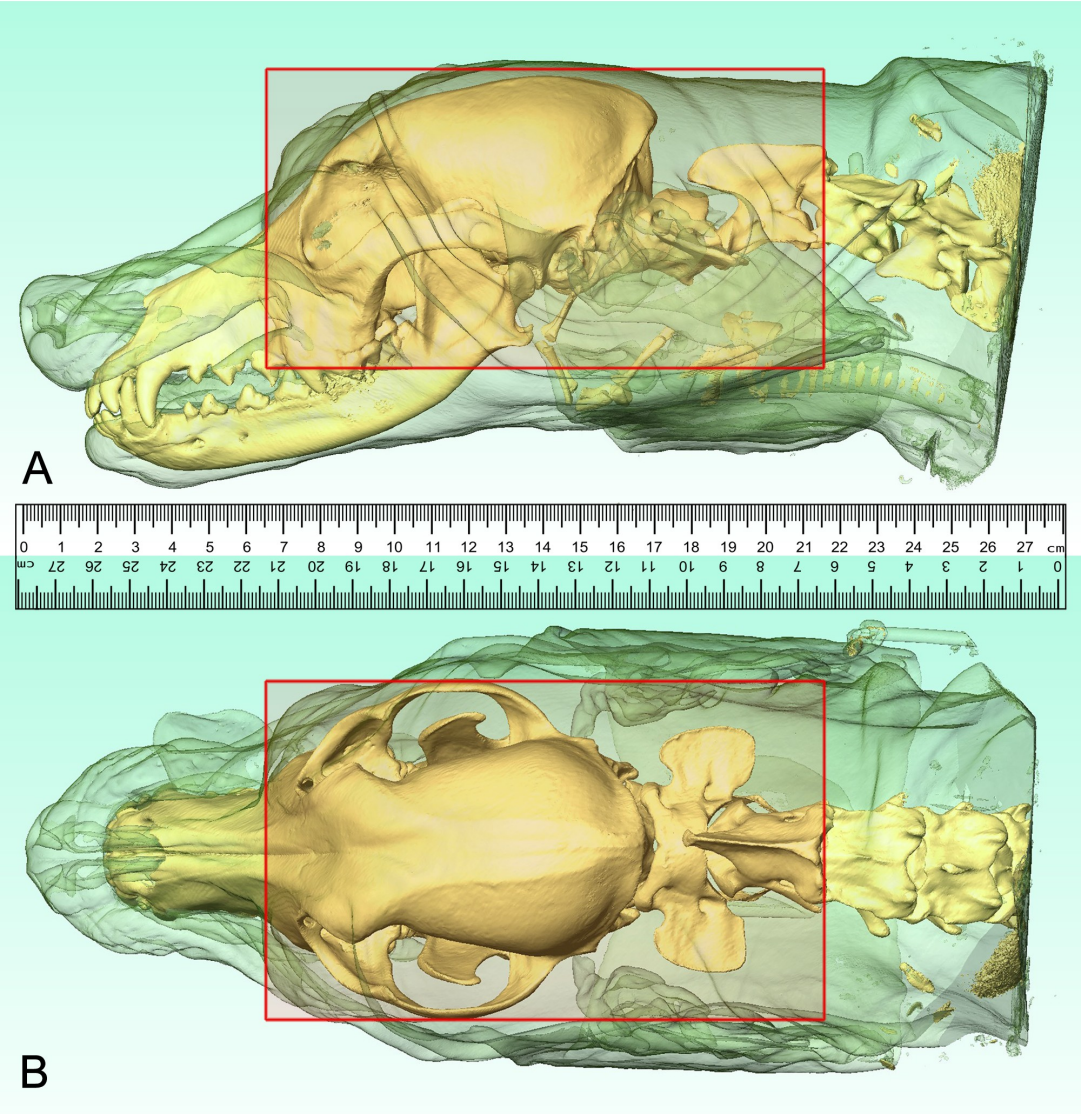
Localizing the boundaries of the head block. Based on the CT-scans, three-dimensional reconstructions were made to determine the borders of the head block (red rectangle). Lateral (A) and dorsal (B) views.

The 3D-reconstructions were made from the acquired DICOM-images using Thermo Scientific Amira for Life Sciences 6.0 software (FEI Visualization Sciences Group, http://www.fei.com). Using the *in-* and *ex vivo* CT and MR images we precisely defined the transverse plane of milling, and the boundaries of the frozen neurocranium block were determined (Fig 1). The horizontal extension went from the level of the infraorbital foramen to the cranial vertebral incisure of the third cervical vertebra (a total of 150.19 mm). The vertical extension went from the highest point of the calvaria till the level of the upper fourth premolar teeth (a total of 80.06 mm). Based on the acquired 3D-models, a head block was made (S1 Fig), which was embedded into a thermoregulated box containing water-gelatin mixture (according to a previous study [22], but without adding methylene blue dye).

### Cryomacrotomisation

The cryomacrotomisation was performed with a Kondia NCT B-640 precision milling machine (S2 Fig). The cut surface was cleaned with 10% isopropyl-alcohol, cooled before each sectioning with a CO_2_ cryo-gun (Linde AG, Germany) and grinded dry ice (3 mm pellets). The embedding box was repeatedly filled with dry ice to achieve sufficient mantle cooling around the block. Liquid nitrogen was also used to cool the block. In order to capture the individual surfaces, we used a Nikon D800 camera and an AF-S VR Micro-Nikkor 105 mm f/2.8G IF-ED lens with polarized filters and color-checkers (ISO-100, focus distance 105 mm, exposition time 1/200 sec, aperture size 3.1, F-stop f/8, with X-Rite ColorChecker passport and polarizing filters). Photos were recorded in 24-bit color depth and 300 dpi RAW pictures, the dimension of each picture was 7360x4912 pixels, which was set to the extent of the neurocranium block. Overall, we recorded 1112 images.

### Image registration

The RGB images were imported into Amira (creating a single volume where voxel size was 19.5x19.5x100 μm), then DICOM images from *in vivo* CT and MR imaging were also brought into the same “Project View” space. Possible shifts between adjacent color images were corrected with the “Align Slices” module, then the whole volume was resampled and JPEG images were exported in all the main orthogonal (“xy”, “xz”, and “yz”) directions. CT images were aligned with the MR images with the “Register Images” module, then the whole RGB volume from the cryomacrotomisation was also fit to the MRI series, thus all the three volumes (MR, CT and cryosectioned RGB images) were in the same coordinate system. Registration steps included rigid and non-rigid transformations. The rigid transformations were rotation and translation, and the non-rigid transformations were isoscaling, anisoscaling and shearing. The metrics used in the Amira during the registration included ‘Correlation’ and ‘Normalized Mutual Information’. The primary data of the registration was the cryosectioned image volume, and the overlay data on it was the bone kernel defined CT-volume. The registration was done automatically by the software. After aligning the centers of the primary and overlay data, first a rigid, then a non-rigid transformation was applied. In the Multiplanar viewer the overlay of the two image sets could be checked in the three main orthogonal planes. Fitting of the osseous structures, which were clearly visible both on the cryosectioned and CT images, was used to check the proper alignment. It also included the inspection of the area of the lamina cribrosa, the frontal sinus, external sagittal crest, external occipital protuberance, tympanic bulla, and the placement of the basioccipital-, basi-. and presphenoid bones. After the registration was successfully completed between the two dataset, the CT series was resampled (with the ‘Resample transformed image’ module). Afterwards, the aligned CT-series was used as a primary data, and the MR-series for the overlay data, and as described previously, rigid and non-rigid transformations were applied to properly align the two datasets. After the inspection of the result, the MR-image volume was also resampled, and thus all the three image volumes were aligned with each other. Using the “Slice” module the same orthogonal views could be set on the three imaging modalities, making them directly comparable in the same position according to the global coordinate system.

### Image segmentation and 3D-modeling

**Fig 2:**
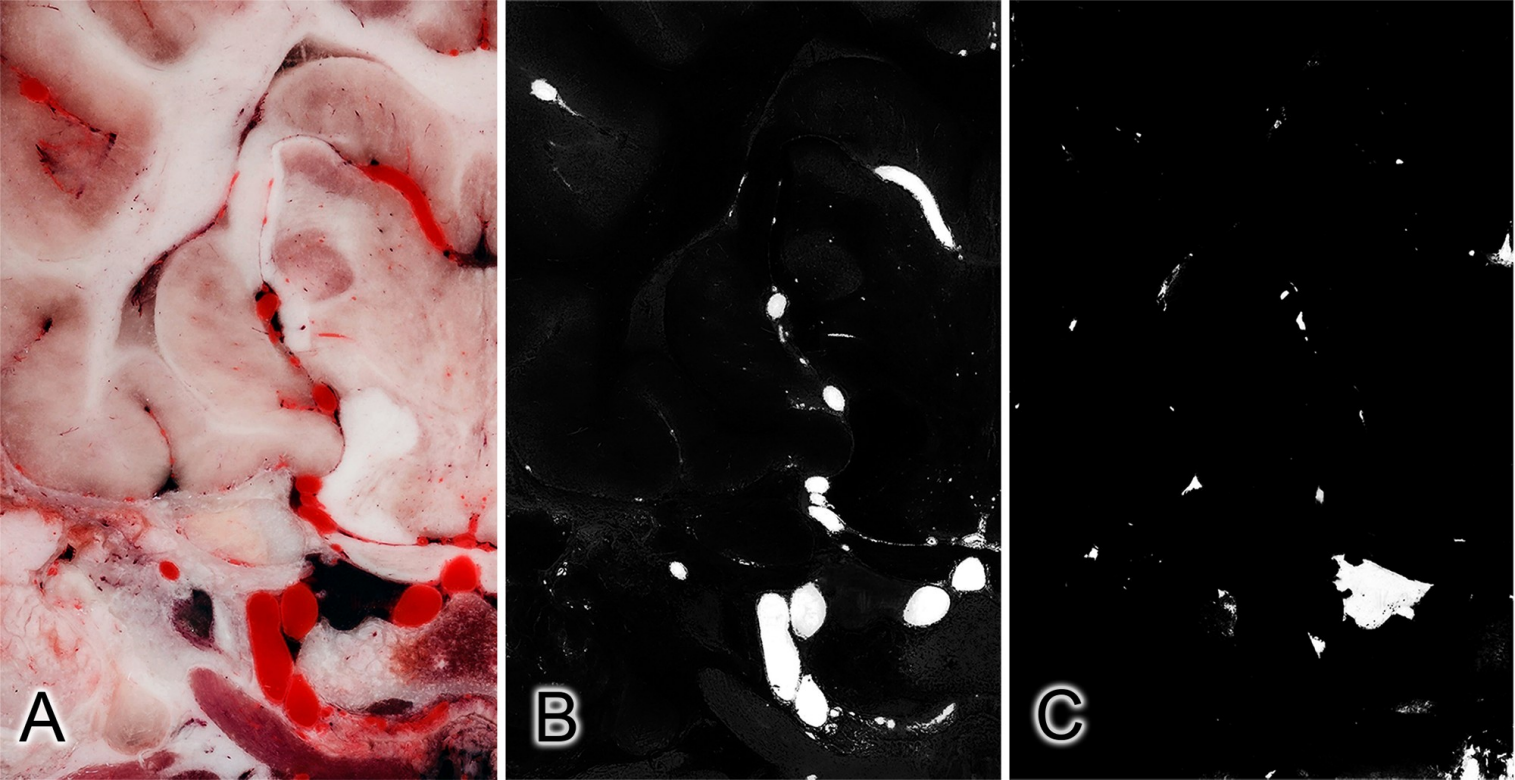
Subtracting the vessels from a cryosectioned image: Effects of the applied filters on the same slice. Original color image (A), and after selective filtering for the arteries (B) and veins (C).

Using Adobe Photoshop CS3, two sets of grayscale images were created from the original RGB-series with selective filtering and enhancing the structures to filter out the arteries and veins for the semi-automatic segmentation (Fig 2). In order to selectively filter the arteries and veins, Photoshop action-files were created; these performed a pre-programmed action-series on multiple images. In the case of the arteries, a ‘High pass filter’ was used to enhance the contrast, the ‘red’ channel was extracted to a separate layer from the RGB image, and selective color reducing carried out on the red channel. Subsequently, a black and white conversion was applied by removing the cyan tones, then contrast was enhanced and brightness was slightly reduced with the ‘brightness and contrast’ module. Finally, the RGB image was converted and saved in a grayscale mode to make the segmentation with Amira possible. In order to select the veins (which were already dark due to the post mortem highly deoxygenated blood they contained, which also made their distinction easier), ‘color balance’ and ‘selective color’ modules were used to increase the tone of the veins. Afterwards a black and white conversion was applied, and the contrast was increased in the ‘brightness and contrast’ module, following a decrease in the gamma correction. Finally, grayscale conversion and saving of the image file was recorded in the action file. After production of the completed two action-sets, the automatic process was started to convert all the 1112 cryosectioned images. The JPEG-image series were imported into Amira, and then the same 19.5x19.5x100 μm sized volumes were generated and aligned with the original RGB-volume. For each grayscale volume an “Edit New Label Field” module was generated, and a manually controlled semi-automatic segmentation was performed in the “Segmentation” area. The brain, bones, arteries and veins were labelled separately. Using the “Generate Surface” module, 3D stereolithography (STL) models were created from each label field. Smoothing and refinement of the STL-models were carried out in Amira and Autodesk MeshMixer (freeware, http://www.meshmixer.com).

## Results

**Fig 3:**
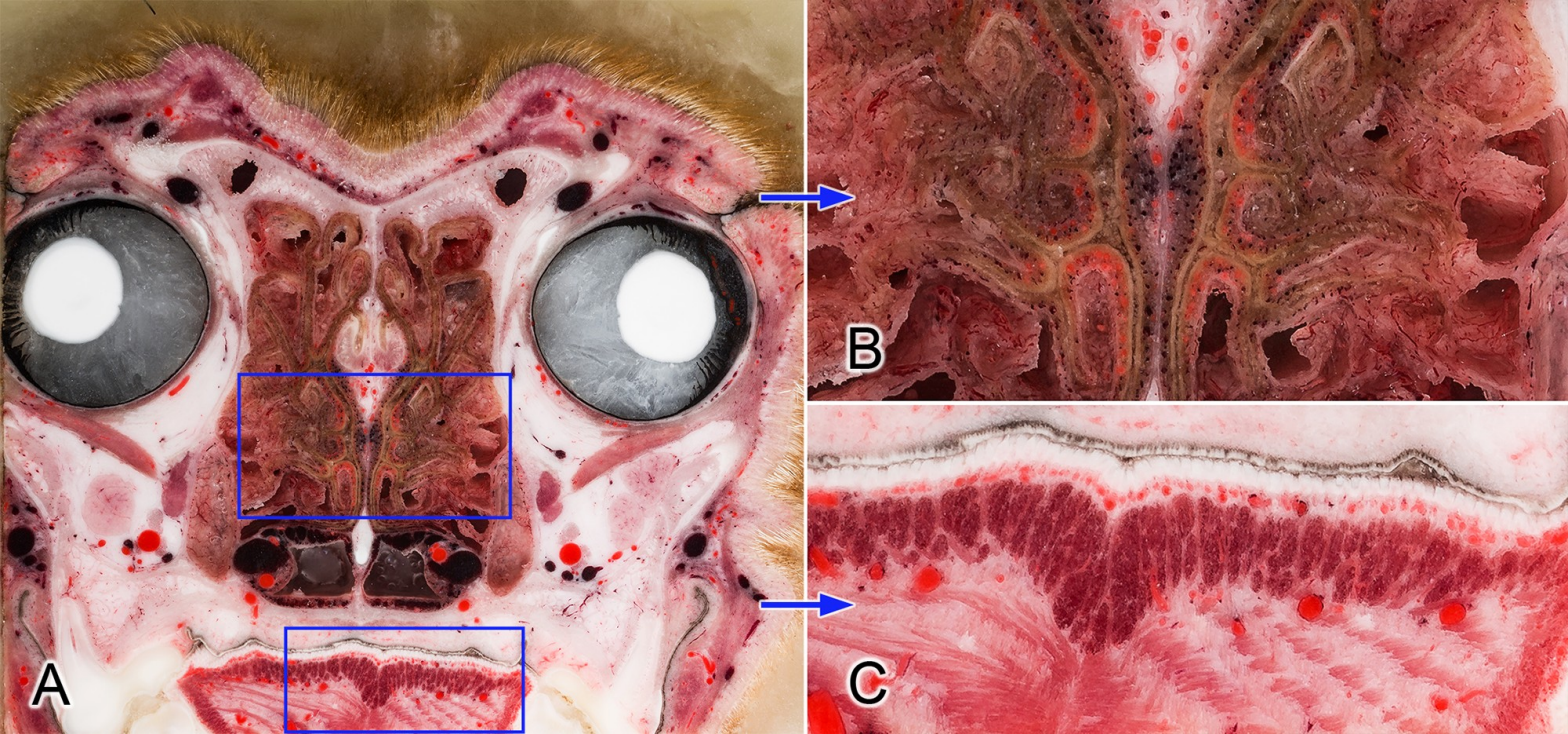
**Transverse section at the level of the orbit (A)**. Blue frames on image (A) show the zoomed regions (B and C). Close-up views show the detailedness of the ethmoturbinates in the nasal cavity (B) and the intrinsic muscles of the tongue (C).

**Fig 4:**
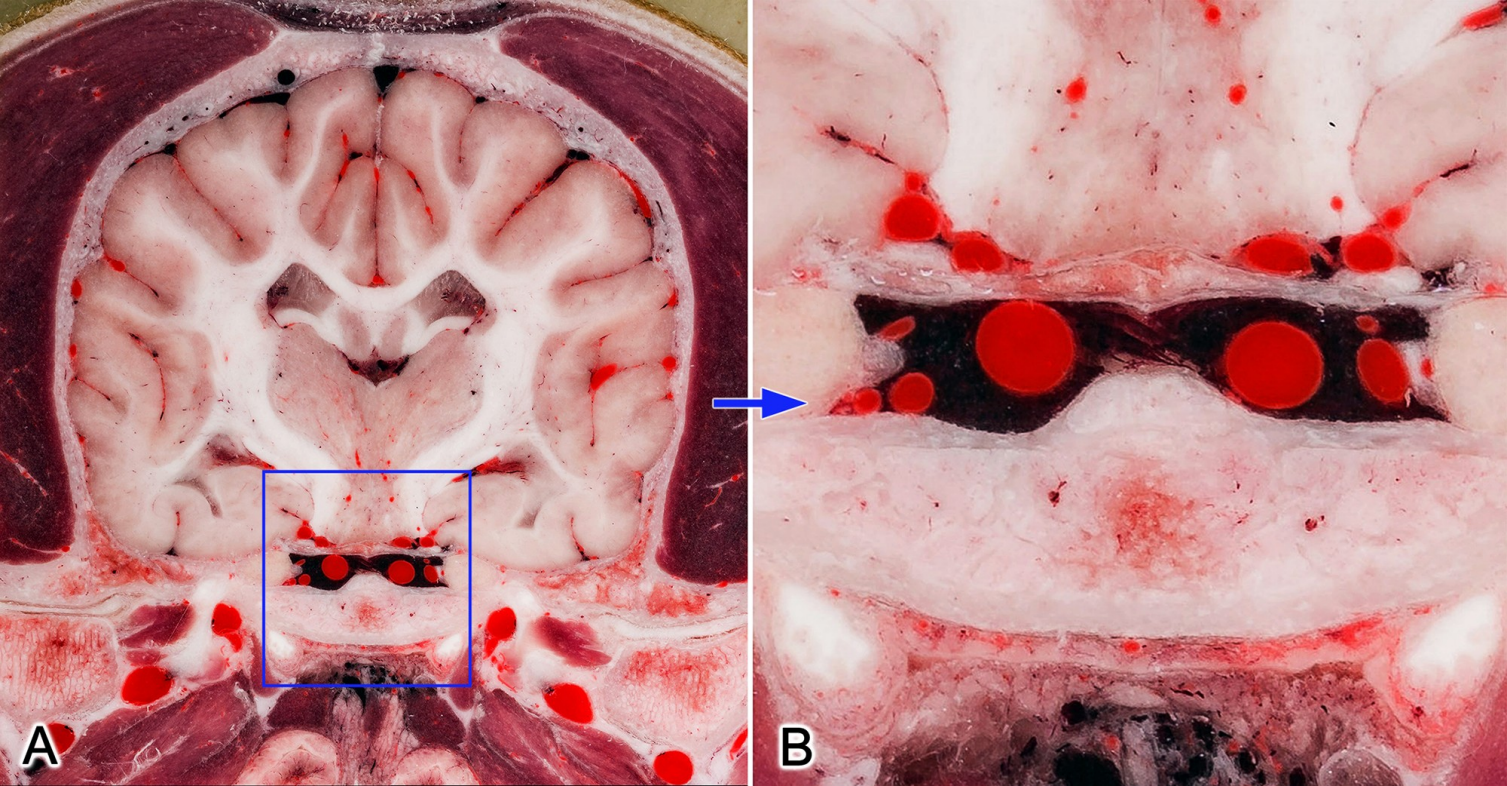
**Transverse section at the mid-thalamic level (A)**. Blue frame on image (A) shows the zoomed region (B). Close-up view (B) shows the detailedness of the vessels (like sinus cavernosus, internal carotid arteries and branches of circulus arteriosus). https://doi.org/10.6084/m9.figshare.c.4365566.v1.

Using high resolution photography, the brain and the surrounding structures were visualized with high acuity and detailedness, as it is shown in examples zooming on the ethmoturbinates, intrinsic lingual muscles and structures of the middle cranial fossa (Figs 3 and 4).

**Fig 5:**
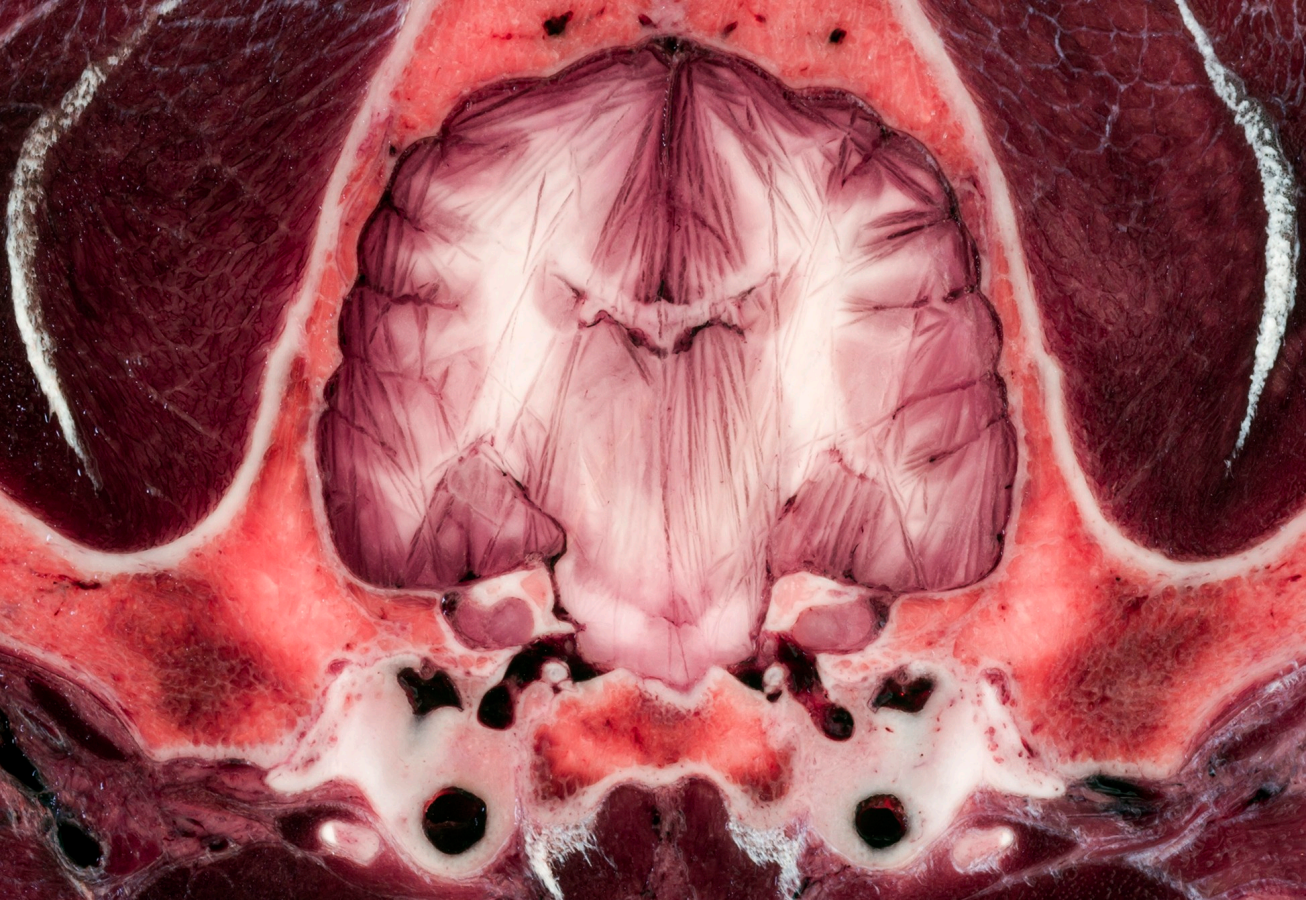
Transverse section of a dog brain showing discoloration and frozen line artefacts.

**Fig 6:**
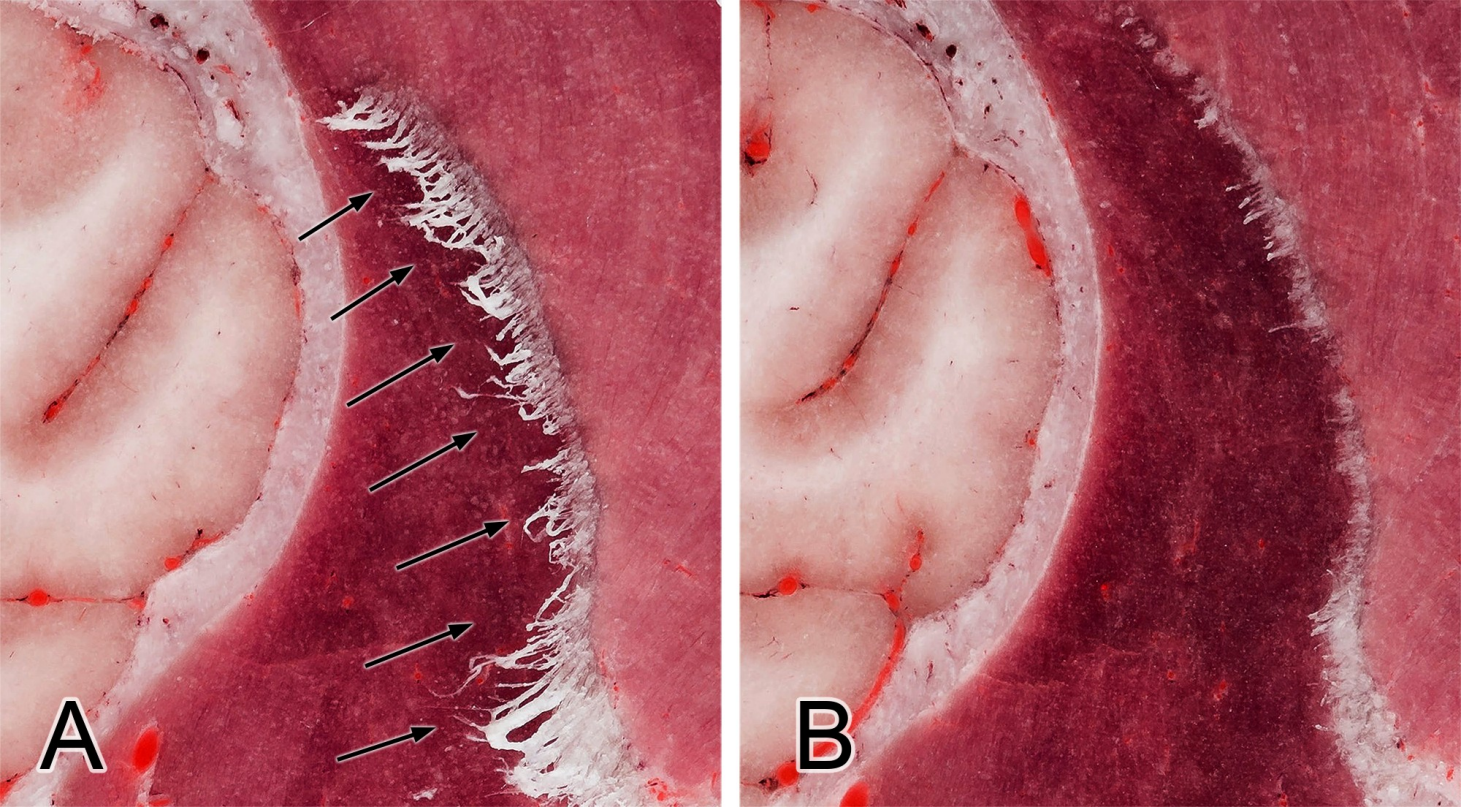
Fiber-formation: Demonstrating the importance of proper cooling. Arrows show the fibrous-tendinous tissue of the temporal muscle attaching on the coronoid process of the mandible (A). After the treatment with dry ice and liquid nitrogen these fibers harden enough to eliminate them during the upcoming sectionings (B).

**Fig 7:**
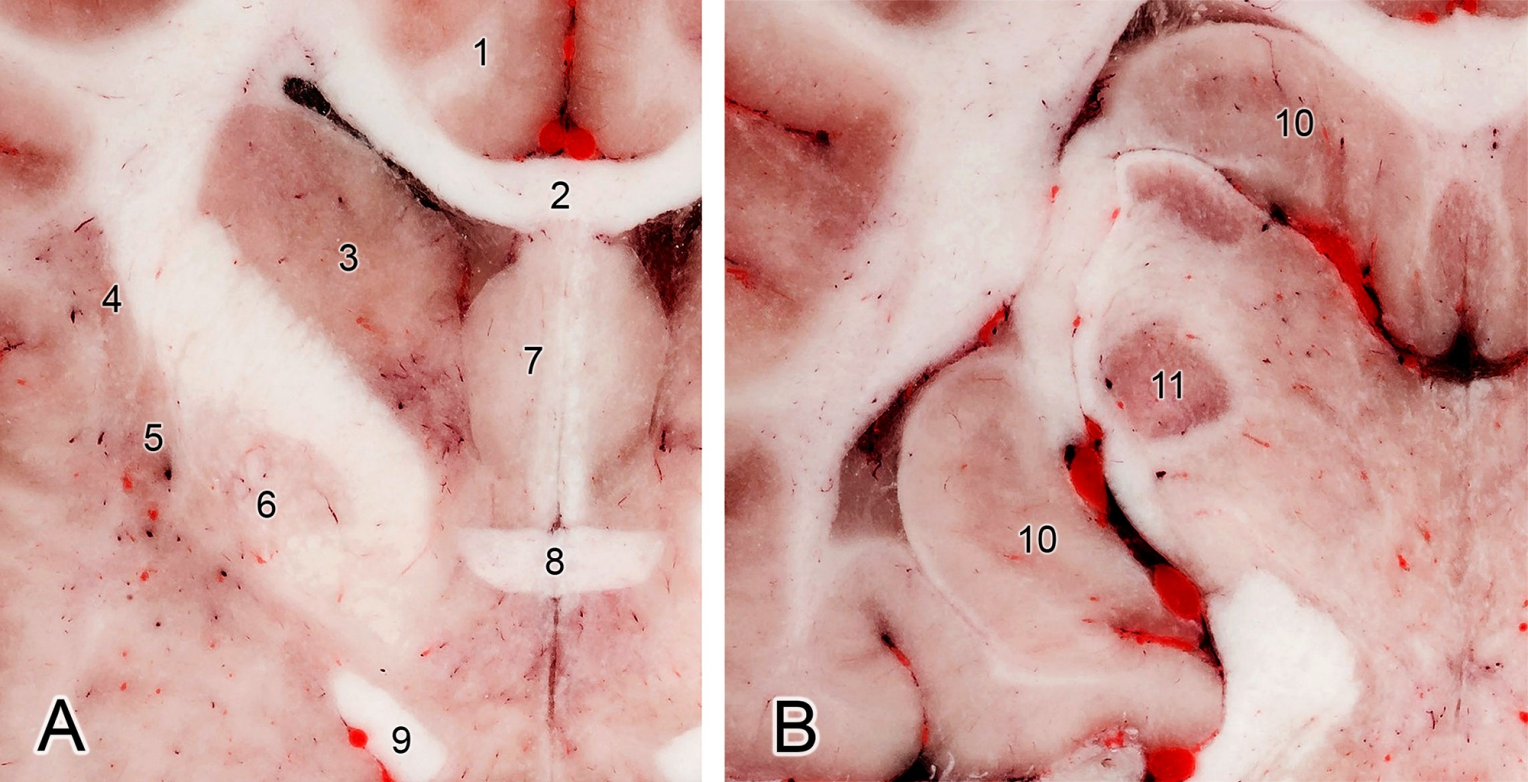
Transverse section at the level of the rostral commissure (A) and at the level of lateral geniculate body (B). (1) Cingulate gyrus. (2) Corpus callosum. (3) Caudate nucleus. (4) Claustrum. (5) Putamen. (6) Globus pallidus. (7) Septal area. (8) Rostral commissure. (9) Optic tract. (10) Hippocampus. (11) Lateral geniculate nucleus.

The known major challenges when cryosectioning the brain could occur during the cooling process. Since the brain contains a large amount of water (approx. 70%) and freezing takes a long time, artefacts can occur. One of this is that rapid cooling of the tissues during the embedding procedure (using liquid nitrogen or dry ice) can result in soft tissue expansion. We could observe this effect during the process, as a small part of the splenial gyrus was moved underneath the tentorium cerebelli membranaceum, and the pyramid of the vermis moved towards the foramen magnum. The subarachnoid space and the encephalic ventricles were also smaller compared to those seen on the MRI. On the other hand, if the cooling process of the block is not properly performed and it takes a longer time to freeze the block, then discoloration of the gray matter and surface artefacts (“frozen lines”) can be seen during the cryosectioning, as we found in our previous study on a cadaveric brain (Fig 5). Another important issue during cryosectioning may occur when fibrous tissues are found in the working area (e.g. tendons, or thicker perimysium and epimysium). These tend to become frayed or fibrous because, if they are not properly cooled prior to sectioning, they are not cut sufficiently by the device (Fig 6A). This phenomenon has also been observed by other researchers [21,23]. If this happens, then a manual intervention is required, e.g. using a scalpel or scissor to trim these fibers. However, if the upcoming layer before milling is appropriately cooled, this ensures optimal results in the cryosectioning (Fig 6B). During the cryomacrotomisation of the brain of the current study we did not see frozen lines on the surfaces, which confirmed that the cooling of the neurocranium block was appropriate, no signs of discoloration occurred, and the fiber-formation was continuously controlled in order to remove possible filaments. The gray matter hence appears in its original reddish color, and even though no tissue staining was applied, the boundary between the gray and white matter and the outline of the major subcortical nuclei can be clearly identified (Fig 7).

**Fig 8:**
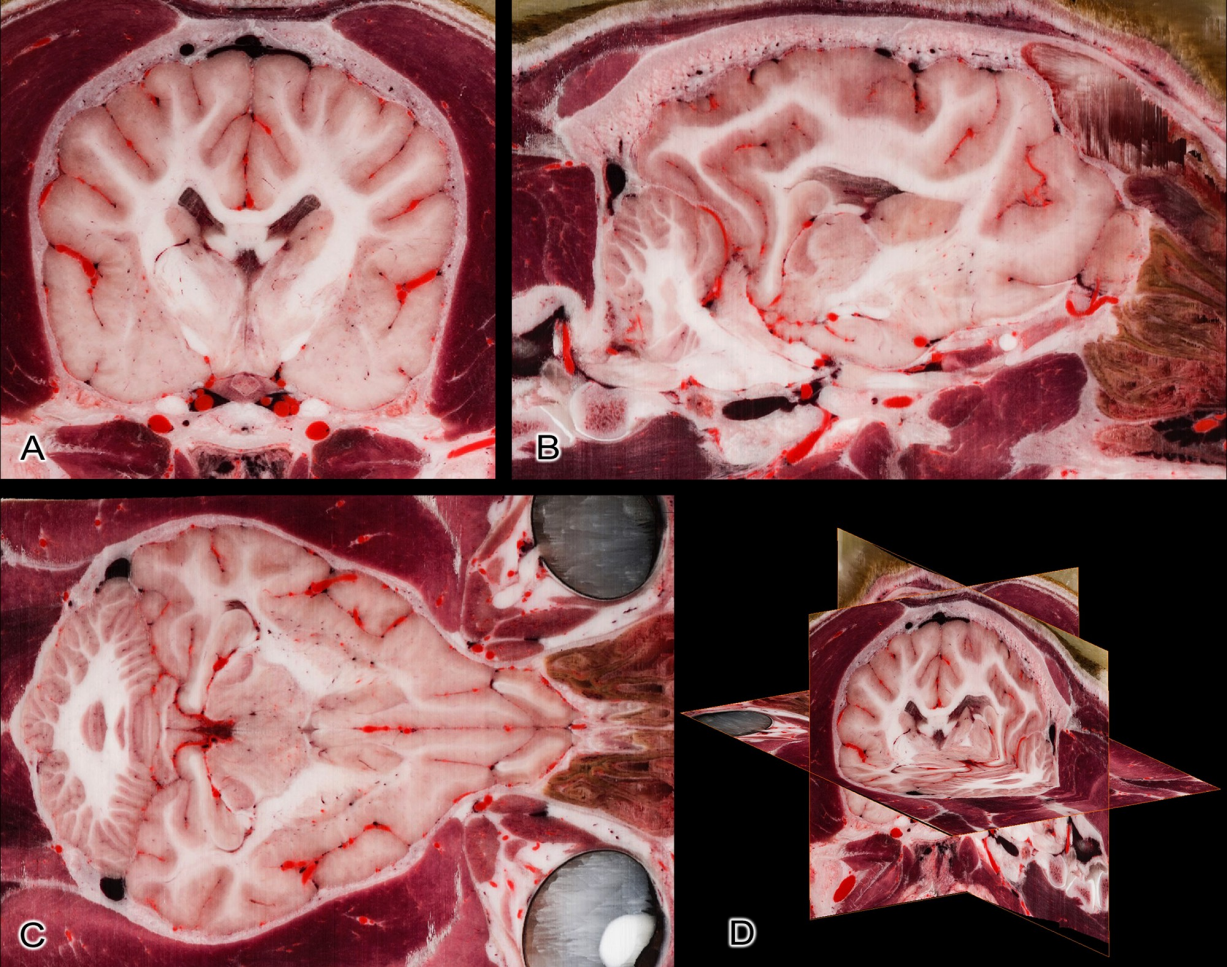
Reconstruction based on the original transverse cryosectioned images. (A) Transverse plane (original image). (B) Sagittal plane (reconstructed image). (C) Dorsal plane (reconstructed image). (D) Three-dimensional composite picture of the orthogonal views.

**Fig 9:**
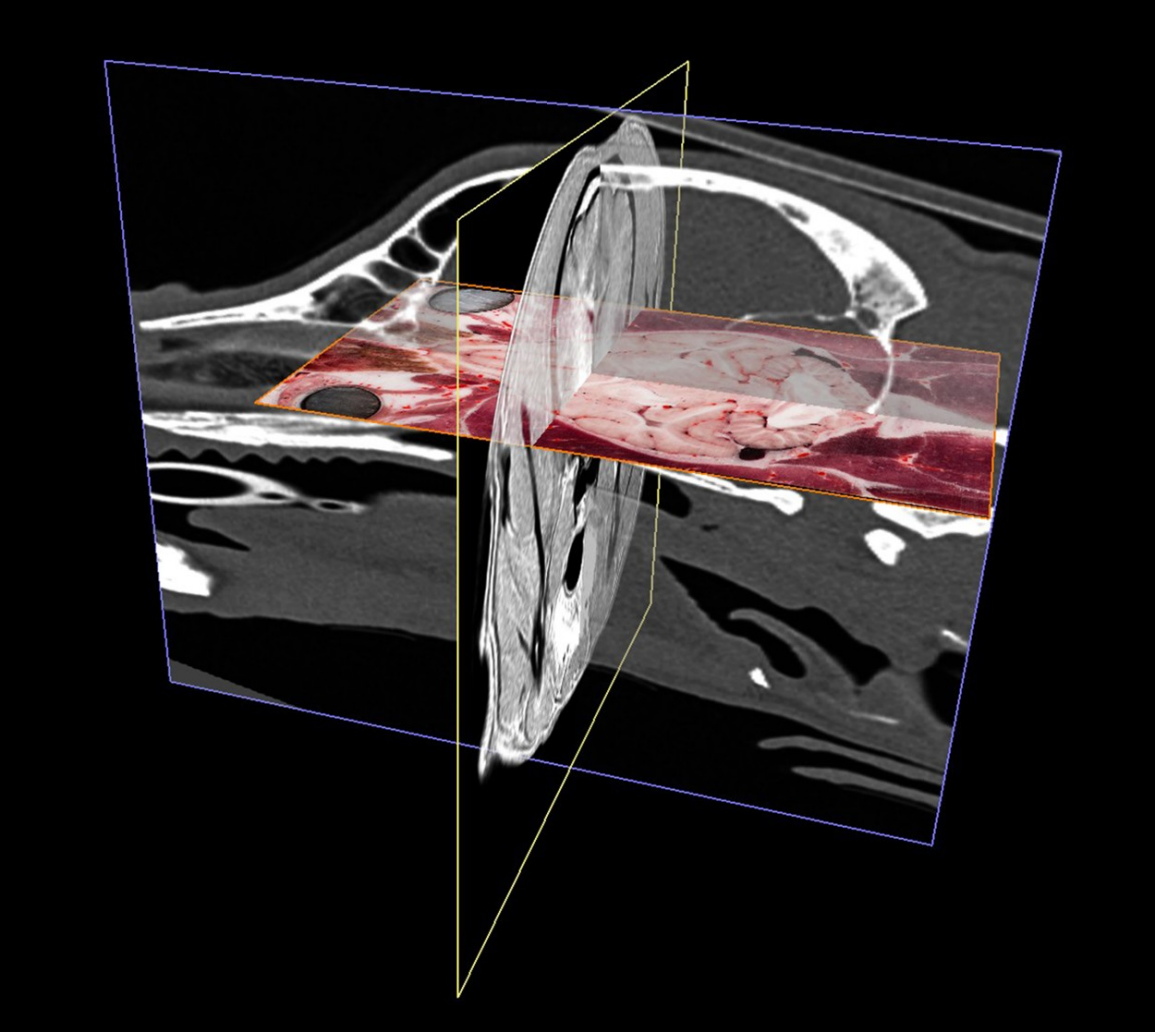
The three co-registered imaging modalities in a perspective view. MR is in the transverse plane (yellow frame), CT is in the sagittal plane (blue frame), cryosectioned image is in the dorsal plane (orange frame).

**Fig 10:**
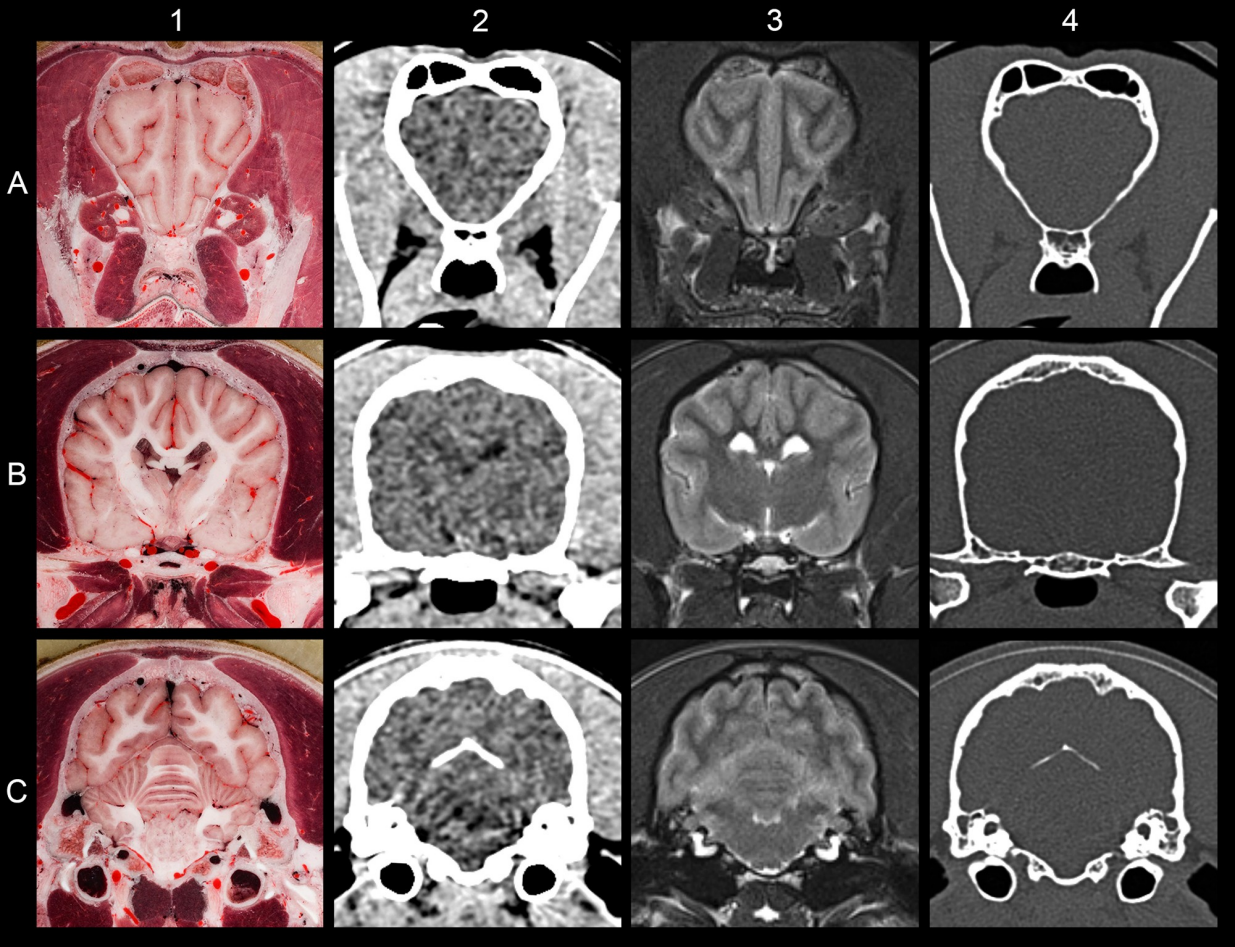
Comparing the result of different imaging modalities at the same level. (A) At the level of the olfactory peduncle. (B) At the level of the hypophysis. (C) At the level of the cerebellum. (1) Cryosectioned image. (2) CT with brain window. (3) T2-weighted MRI. (4) CT with bone window.

The relative small (100 μm) slice interval made it possible to reconstruct the other orthogonal (sagittal and dorsal) planes without losing the detailedness of the individual structures (Fig 8). This means that even on a higher magnification the structures of the computer-reconstructed slices appear as sharp, detailed and uninterrupted, as if the cryosectioning had occurred along that plane. Registering the CT and MR images to the cryosectioned volume resulted in a nearly perfect comparison between the different imaging modalities. Any possible biases from the original position are the result of the mild expansion of the tissues during the freezing process. Images from various sites show this good multimodal comparability (Figs 9 and 10).

**Fig 11:**
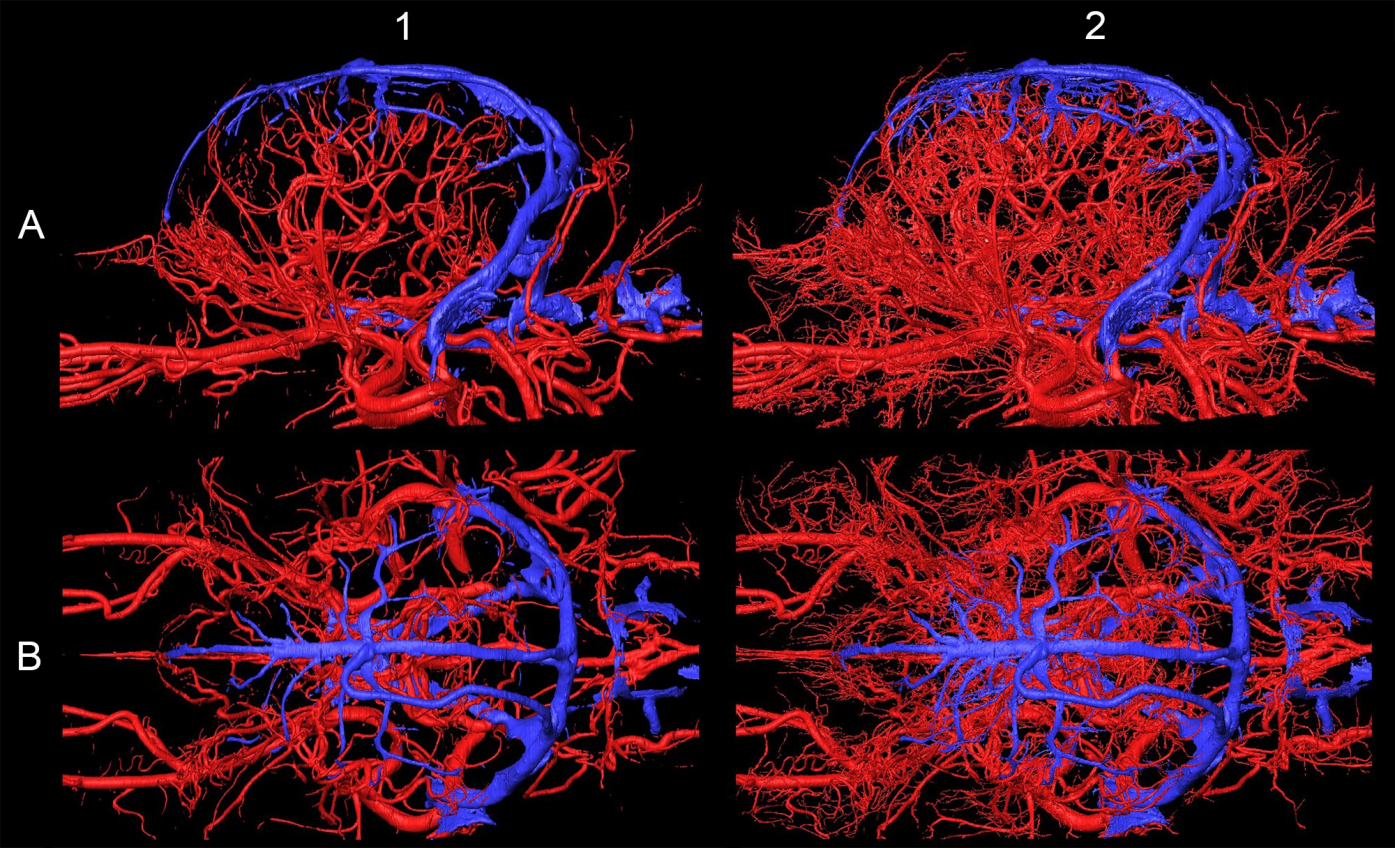
Arteries (in red) and veins (in blue) on the 3D-model with different detailedness. (A) Lateral view. (B) Dorsal view. (1) Less detailed 3D-model. (2) Highly detailed 3D-model.

**Fig 12:**
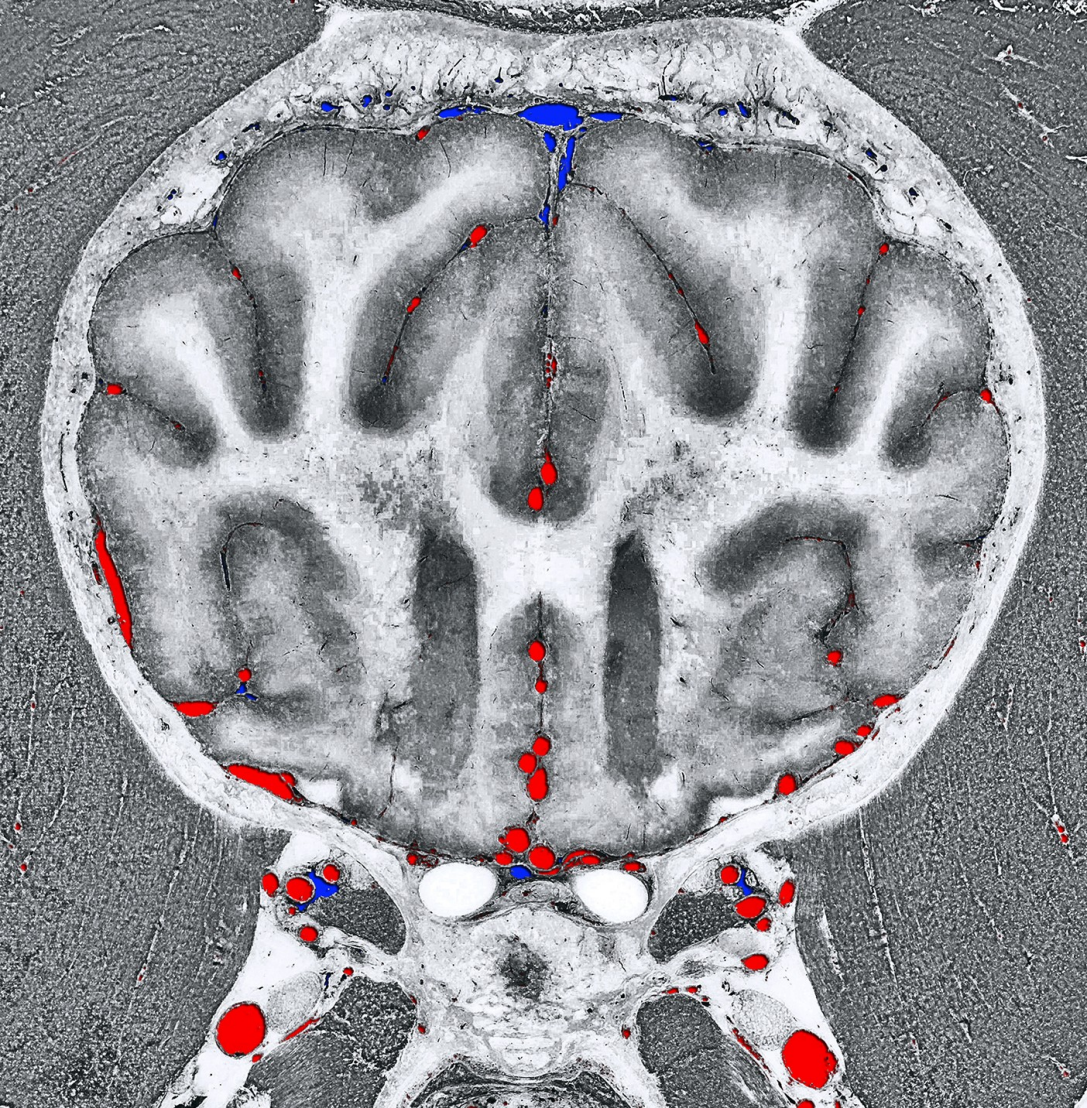
Transverse section at the level of the frontal lobe. Composite image with the grayscale-converted cryosectioned image and the crossing vessels from the 3D-models (arteries with red color and veins with blue color).

**Fig 13:**
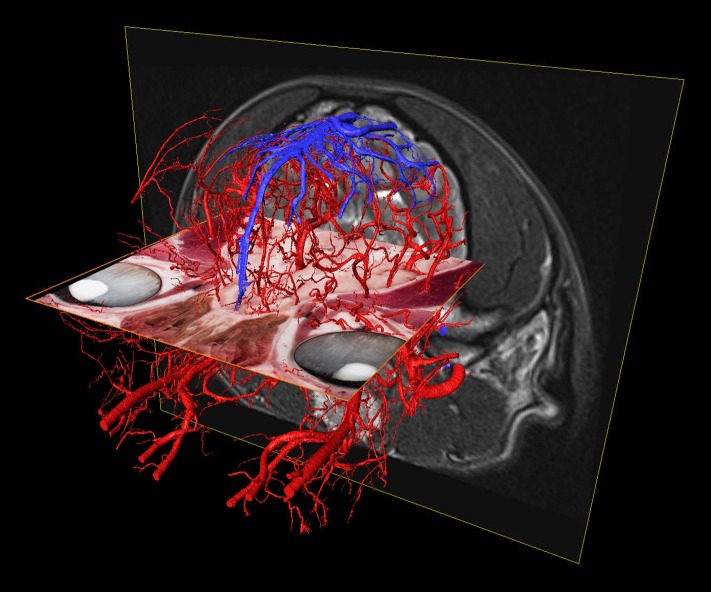
The different imaging modalities together with the vascular 3D-models. Composite image with the cryosectioned layer (dorsal plane, orange frame), the T2-weighted MRI (transverse plane, yellow frame), and the 3D-models of the major arteries and veins. Left rostro-lateral view.

**Fig 14:**
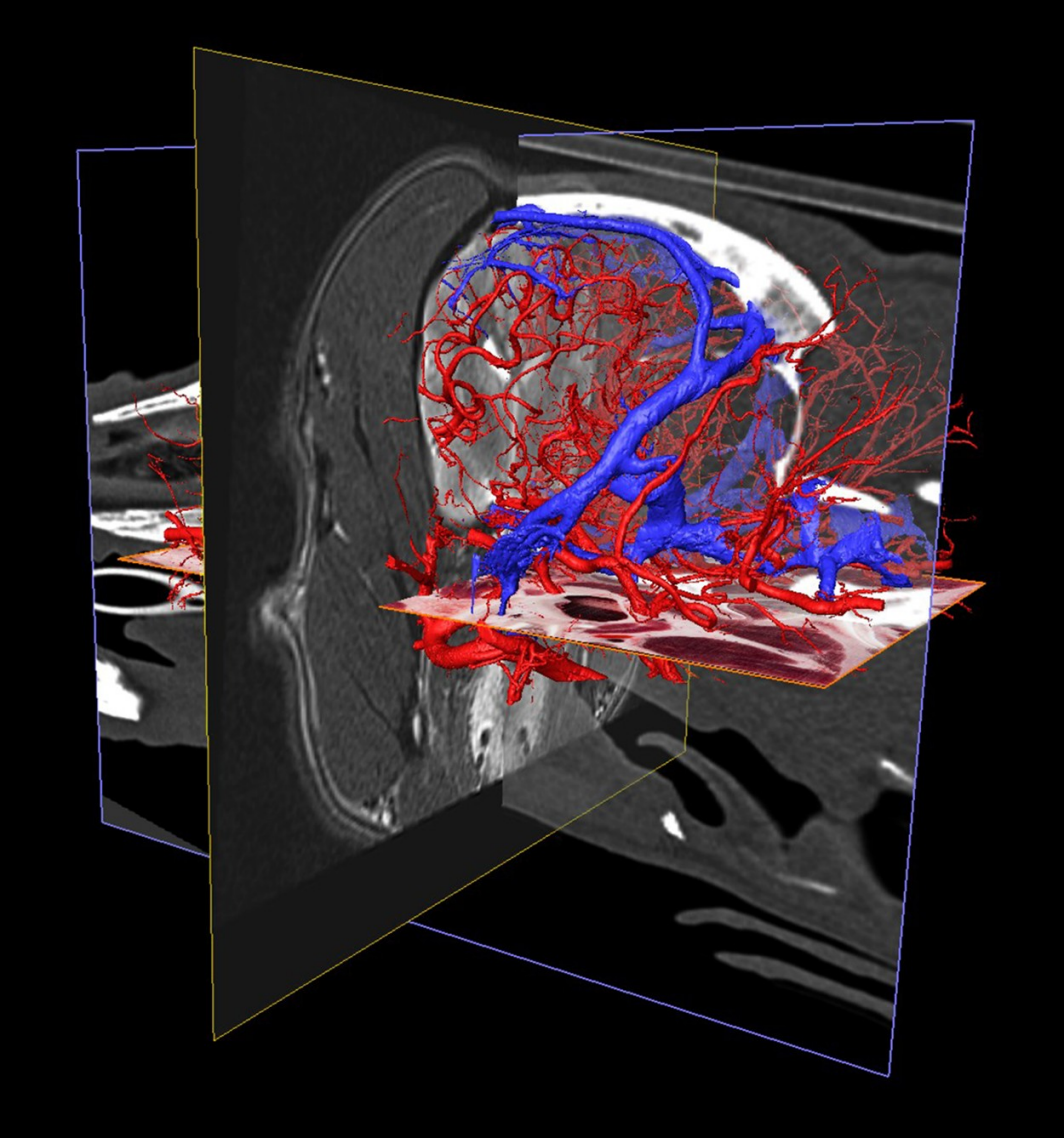
The different imaging modalities together with the vascular 3D-models. Composite image with the cryosectioned layer (reconstructed in dorsal plane, orange frame), the T2-weighted MRI (transverse plane, yellow frame), CT-scan (sagittal plane, blue frame) and the 3D-models of the major arteries and veins. Left caudo-lateral view.

As arteries and veins were segmented from the cryosectioned image volume, separate 3D-models were generated and transferred into a common space, showing the difference between highly detailed segmentation and focusing only to the main branches (Fig 11). This can be best seen at the area of the lamina cribrosa, where the caudal septal nasal arteries pass into the nasal cavity: on the B1 part of Fig 11 only the main contour of the ethmoidal fossa is highlighted by the larger vessels, but on B2 the small nasal branches are also visible. As all the image volumes (cryosectioned images, MR and CT series) and the surface models were registered in the same coordinate system there were several visualization possibilities. They could be shown in any association with each other, thus allowing easier identification of the vessels on the grayscale cryosectioned images (Fig 12), or by determining the position of the arteries and veins (according to their 3D-models) the tracking on the MR and CT imaging could be more straightforward (Figs 13 and 14).

**Fig 15:**
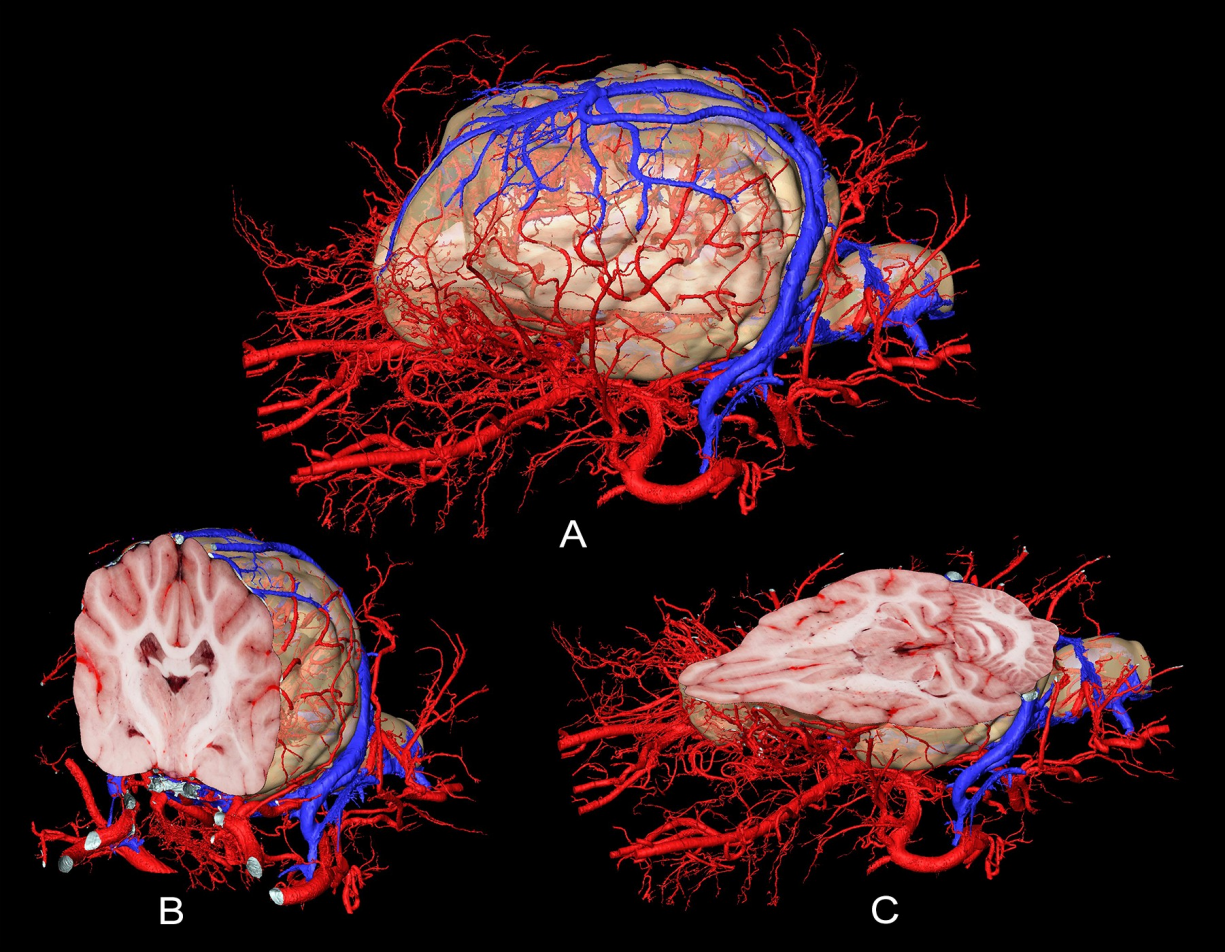
Surface model of the vessels, and the extracted brain model. Models as a whole (A), and sectioned in the transverse plane (B) and the dorsal plane (C). Arteries with red, veins with blue color.

**Fig 16:**
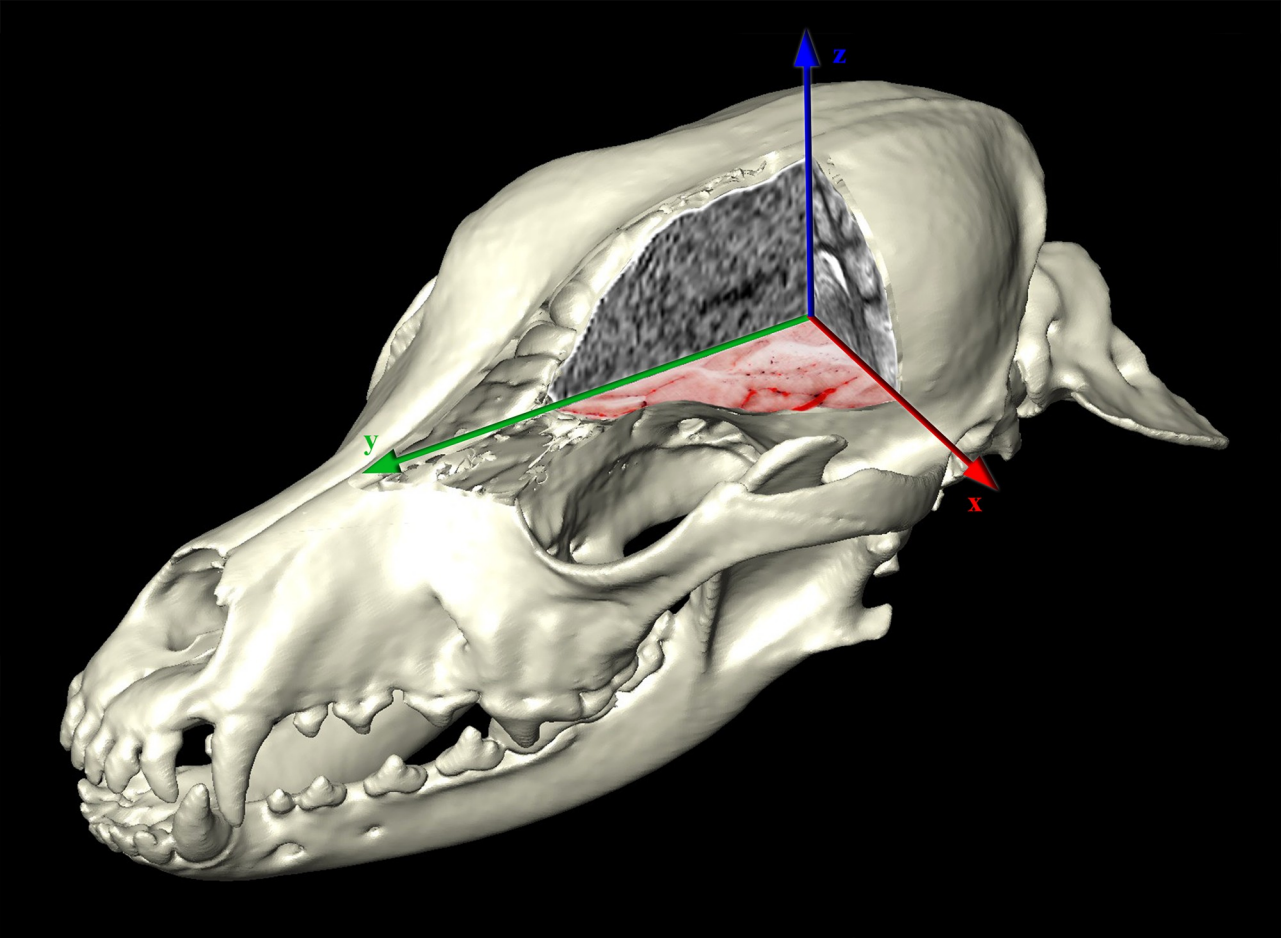
Surface model of the skull with the three main imaging modalities presented in the different orthogonal planes. Cryosectioned image is on the dorsal (“xy”) plane. MR image is on the transverse (“xz”) plane. CT image is on the sagittal (“yz”) plane.

Finally, to give further perspectives for the possible use of this kind of dataset, the interactive use of the softwares allowed us to dynamically examine the subtracted volume rendered 3D-models (cut in any arbitrary plane) in order to show only the brain and its supplying vessels from different point of views (Fig 15). By setting a relative starting point (choosing any point inside the volume), all the three main imaging modalities can be visualized in different planes with the 3D-model of the skull (Fig 16). With the latter method, immediate comparison of the neurocranial structures is possible by changing the slice number and switching between the planes and imaging modalities.

## Discussion

One of the main advantages of cryosectioning is that any region of the body can be studied, regardless of the tissue composition including thick bones that can be easily cut. Therefore, high resolution RGB-images can be obtained, and if the layer thickness is small enough, multiplanar reconstructions (MPR) can be made in any arbitrary plane by using specially designed softwares (e.g. Amira or 3DSlicer). When structural imaging techniques (MR, CT) are associated, the volumes can be registered together, thus multimodal atlases can be easily generated in the same coordinate system. As ante mortem functional studies, tractography of neural pathways (Diffusion Tensor Imaging, DTI), PET (Positron-Emission Tomography) and SPECT (Single Photon Emission Computed Tomography) imaging could also be done prior cryosectioning. The segmentation of individual structures or systems makes it possible to create three-dimensional models beneficial to several fields, including education (e.g. understanding the relationship between the 2D images series and the 3D-structure), research (comparative neuroanatomy) and clinical work (reference atlas for studying the conservative brains regions during surgical planning). As in the current study the brain was not removed from the neurocranial cavity, which is usually required to allow histological or macroscopic slicing [42,45], the cryosectioned structures can be seen in their original position (depending on the occurrence of freezing expansion or shrinkage). For example, the vessels around the brain and the nerves, which are entering and leaving the skull through different foramina can be traced around the osseous structures and the central nervous system. As no fixative agent was used in our study, and only the arteries were filled with red polyurethane (without producing extravasates), all the tissues can be seen in their original color. This was also confirmed by using a color checker passport, which helps with camera and image calibration during the post-processing procedure of the cryosectioned images.

The time required for the entire process can be divided into three main parts: the pre-sectioning, sectioning and post-sectioning work. (1) The pre-sectioning workflow includes the implementation of the diagnostic imaging techniques (e.g. CT and MRI), freezing the body and blocking the region of interest, constructing the embedding device, establishing the angle of cut in accordance with the imaging techniques orthogonal planes (so the milled surface could be parallel with them) and embedding the block. (2) The cryosectioning part comprises the preparation of the milling device, the cooling-, camera- and computer systems, and taking the images. (3) The post-sectioning part covers the photography post-processing (e.g. setting the proper color balance based on the color checker, equalizing shadows, and generating JPEG or TIFF images from the raw CR2 image series), and depending on the aim of the study, the image analysis or 3D-modelling (which consists of the volume-registration, segmentation, and 3D-modelling work at the required level of detailedness). The time of workflow is affected by the number of people who contribute to the research (e.g. in the case of multiple species, organizing a parallel work to progress is advised), the stops during the cryosectioning (in our case the milling procedure of the canine neurocranium took approximately 40 hours). The pre-sectioning work required two weeks, photo-conversion of the images at the post-sectioning process took three days. The post-sectioning image analysis and 3D-modelling work solely depends on the research goals (e.g. number of modelled structures and their detailedness), and the number of people working simultaneously on a project.

Known technological limitations are mostly dependent on the milling device, and the cryogen process itself. There is no technical limitation regarding the species, as any tissue can be sectioned by the milling device, which was originally designed to work with metals (thus, even sites with surgical implants could be cryosectioned). If the block is too large it can be divided into smaller parts, as it was done during the Visible Human Project. Imbalanced cooling could cause artefacts, which include discoloration, surface frozen lines or expansion/shrinkage of the tissues, as described before. The 100 μm layer thickness can be still reduced, as the milling device is capable of working with a 1 μm precision with metals. However, the expansion (due to the heat originating from the milling) and the refreezing (after capturing the surface, prior to the next sectioning), could cause not only temperature-fluctuations, but uneven freezing and discoloration of the surface (based on tissue quality) can also occur. Thus, going below 20–30 μm can lead to challenges with the cryomacrotomisation process, if no prior tissue fixative and/or cryo-protective agent is used. Due to the fact that the block is destroyed during the milling procedure (the device “carving down” the block layer by layer), the sectioned tissue cannot be used for other purposes after the cryomacrotomisation. Theoretically, the resolution achievable can be nearly unlimited, as with a high quality DSLR camera sensor one could not only capture the overall surface, but also the surface could be subdivided into four, twenty or hundreds of smaller regions to be captured individually. Thus, one surface can be merged from several regions, so the resolution of the final image (even with the same camera) will increase. Consequently, the pixel per centimetre ratio could be increased, and is only limited by the actual intention of the researcher. During the post-processing work the limits of the segmentation depends on: (a) the original image resolution (the smaller the pixel to μm ratio, the smaller the vessel that can be identified); (b) the contrast between the vessels and their environment (thus the brightest and the most non-tissue colored resins should be used); (c) the efficiency of the vascular filling with the polymer used; and finally (d) the computer’s capacity, as the hardware should be capable of dealing with larger datasets (e. g. tens of gigabytes) during the computational process.

Compared to other projects that aimed to section dogs [23,40], and a cat [41] we obtained a higher resolution and detailed RGB image series from the region of interest (ROI): (a) we focused entirely on cryosectioning the neurocranium with the brain, whilst other authors sectioned and captured entire cadavers, therefore our ROI for the head was smaller; (b) our slice interval was 0.1 mm compared to 0.2 mm [23,41] or 1 mm [40]; (c) the resolution of our images were higher, 7360x4912 pixels compared to 5616x3328 pixels [23,41] or 1928x1459 pixels [40]; (d) the pixel size was 19.5x19.5 μm compared to 100x100 μm [23,41] or 180x180 μm [40]; (e) we only used coloring agent for the arteries, which did not affect the other tissues compared to the formalin-fixation method used previously [40]; (f) discoloration of the brain or frozen lines inside did not occur compared to other studies [41].

When comparing the current work with canine brain and head atlases, some show the histological aspect of the brain, thus an *ex situ* slicing was made and tissue staining was applied [42,46–50]. During our study we did not use any tissue staining, except for coloring the arterial system, so beside that all the tissues show their original color, and still the outline of the gray matter and position of the major subcortical nuclei can be distinguished (as seen in Fig 7). We wanted to show the brain in its *in situ* position, where the surrounding vessels and the cranial nerves, the skull and associated structures can be traced. Other studies have made structural imaging of the canine head and brain: from just a few sections of the canine head and brain for demonstrational purposes with MRI [51–55], or with CT [56,57], ultrasonography [58,59], to detail anatomical marking on the MRI images [47,60,61], using both MR and CT imaging of the brain [62], and the creation of different MRI brain templates from breed-averaged data [63,64]. The brain was removed to section without performing previous diagnostic imaging in two studies. In the first, 4 mm thick transverse and sagittal plastinated slices were made from the brain [65]. In the second study, surface photographs and 18 transverse slices were created following formalin fixation [45]. The advantage of making a cryosectioned series from a single individual who underwent prior diagnostic imaging is that these images are directly comparable to each other. Due to the small slice thickness and the fact that the image volume can be reconstructed in any arbitrary plane, it could also serve as a visual aid to assist in the interpretation of previous studies if the proper slice is chosen from the Beagle brain series. Studies that made both diagnostic imaging and tissue sectioning of the canine head and brain include: (a) head CT with *in situ* cryosectioning of the entire head, with a slice thickness of 8 mm [66]; (b) CT made with an *ex situ* brain slicing, creating 17 transverse sections with a 5 mm interval [67]; (c) *in situ* formalin-fixed sectioning with 9 transverse head sections (from which four included the brain), with two transverse and one sagittal MR images [68]; (d) *ex situ* formalin-fixed and stained sectioning with previous CT-imaging, which resulted in 9 transverse sections of the brain [69]; (e) *ex situ*, formalin fixed and histologically stained brain sections with previous MR-imaging, producing 12 transverse sections (thickness were 3 to 10 millimetres), and three of the brain slices were correlated with the MR images [70]; (f) *ex situ* formalin-fixed brain slicing with prior MR and CT imaging, where 9 transverse, 4 dorsal and 3 sagittal images were compared between the three methods [71]; (g) *in situ* formalin-fixed sectioning of the entire head, with previous CT and MR imaging, creating 18 transverse sections of the entire head, which comprised 8 transverse sections where the brain could be seen [72]. When comparing our dataset to these, the advantages and novelties of the current cryomacrotomisation study are: (a) we used only one dog to obtain all the diagnostic and cryosectioned images; (b) we performed both CT- and MR scanning; (c) we repeated the MR imaging post mortem to have the basis for comparing ante and post mortem changes; (d) we did not use formalin fixation or dyes in order to show the original tissue color; (e) there was no need for the removal of the brain for sectioning, or decalcification of the bones; (f) focused the entire cryosectioning process and the photography only on the neurocranium; (g) used image capturing with a high level of detailedness (24-bit color depth, 300 dpi, 7360x4912 pixels and a pixel size of 19.5x19.5 μm); (h) obtained sectioning interval was only 100 μm; (i) originally 1112 sections of the neurocranium were made in the transverse plane, but due to small voxel size with the software-based volume-rendering, detailed images in any other orthogonal or oblique plane could be created (as Fig 8 shows), or resample with the required slice interval; (j) the registration of the cryosectioned volume with the MR and CT-data could provide directly comparable images (Fig 10); (k) the segmentation of anatomical structures and 3-dimensional reconstruction can be carried out, used on their own, or can be merged with the 2-dimensional imaging data; (l) after normalization to another image volume, the results are comparable with previous studies, and subsequent imaging surveys could be integrated (e.g. matching with a cryosectioned brain when cryo-protection is used).

Development in conventional image analysis and 3D-image fusion is essential as diagnostic imaging methods are gaining more importance in small animal veterinary medicine [73,74] and in fMRI studies [75]. The brain of the dog as a species was previously examined with CT and MRI in several journal publications during the last decades [51,52,60,76,77], it was compared in textbooks [78–80], and histological [42] and diagnostic imaging atlases [61–64,72,81]. Furthermore, due to similarities in development, aging and comorbidity between humans and dogs, investigations have been recently focused on the canine central nervous system [63,82–85]. Veterinary educational modules are also published recently to help graduate learning [86,87]. Compared to those, one of the advantages of our methodology in 3D-modeling is that the structures which could be created are more realistic due to the detailedness and surface morphology because of the original high-resolution cryosectioned images. The knowledge gained by making this study was also successfully used while planning of a transsphenoidal brain surgery in the case of a dog with pituitary adenocarcinoma [88]. These researches and interests highlight the importance of these type of studies, that allow direct comparison with the most up to date diagnostic imaging methods and histological atlases.

## Conclusions

The improved method utilized for cryomacrotomisation in the current study has proved to be successful to serve as a reliable base for a comparative, multimodal brain imaging atlas. As the cryosectioning procedure is not equivalent with a histological study, but it represents a macro-anatomical cross-sectional approach, the desirable resolution for future studies depends on the aim of the study: we believe that the resolution we used is enough for reliable comparison with the CT and MRI series, and for the segmentation of major anatomical structures. When investigating smaller structures and thinner layers (below 20–30 micrometres) we recommend the standard histological procedures. This is the first study in dogs that has visualized the brain using comparative imaging modalities resulting in excellent quality and detailedness (high resolution images with 0.1 mm layer thickness). Possibilities also include the application of 3D-modeling and 3D-printing to enhance learning in graduate and postgraduate studies. In the results section we showed the main advantages of the improved cryosectioning technique, and gave several examples through multiplanar reconstructions how it can be an aid in the comparative imaging by merging the diagnostic imaging modalities (CT, MR) with cryosectioned images and three-dimensional models. We also plan to use cryo-protective agent before freezing the block in future works. Due to the detailedness of the images, the image sets are suitable for selective structure extraction and exact segmentation, as was shown with the skull, brain, arteries and the veins. Thus, the resulting models can be exported, zoomed into and studied in any perspective, which would be an excellent support to accompany textbooks on the subject matter. We are also considering the fact of inter-subject variability would be good to complete these studies results, thus obtaining cryosectioned image series from dogs with dolichocephalic, mesocephalic and brachycephalic skull types are advantageous. Finally, the cryosectioning technique used, provides a unique tool for examining any parts of the body, no matter the hardness of the tissues. As a result, future works can use this technique in order to provide helpful material for educational, scientific and medical purposes.

## Supporting information

S1 Fig The embedding box (A) and the frozen head block inside the holder (B). (TIF)

S2 Fig The cryomacrotomisation process. (TIF)
