# Supplementary figures and images for: MRI, CT and high resolution macro-anatomical images with cryosectioning of a Beagle brain: Creating the base of a multimodal imaging atlas
Source: PLoS One. 2019 Mar 7;14(3):e0213458. doi: 10.1371/journal.pone.0213458 (PMC6405067; doi:10.1371/journal.pone.0213458)

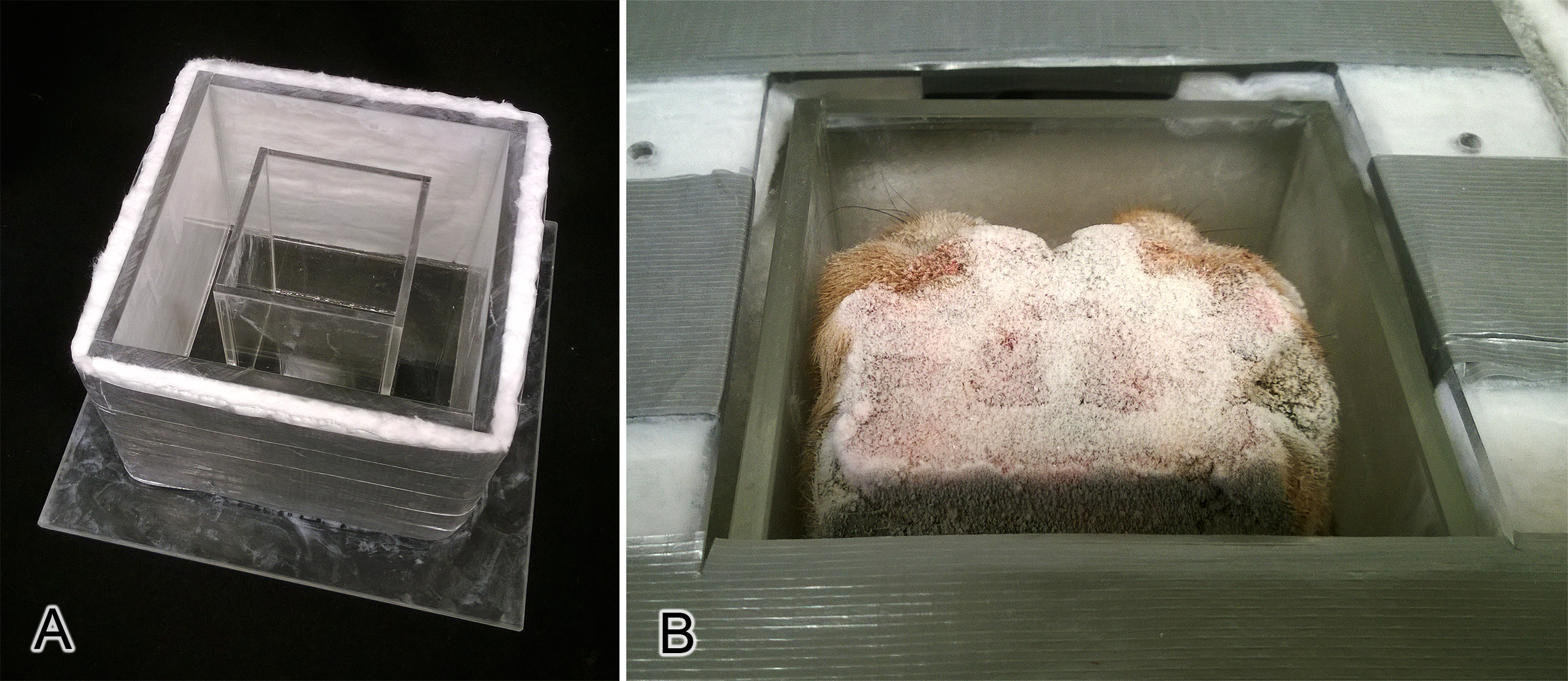

Supplement: S1 Fig — (TIF) [file pone.0213458.s001.tif]

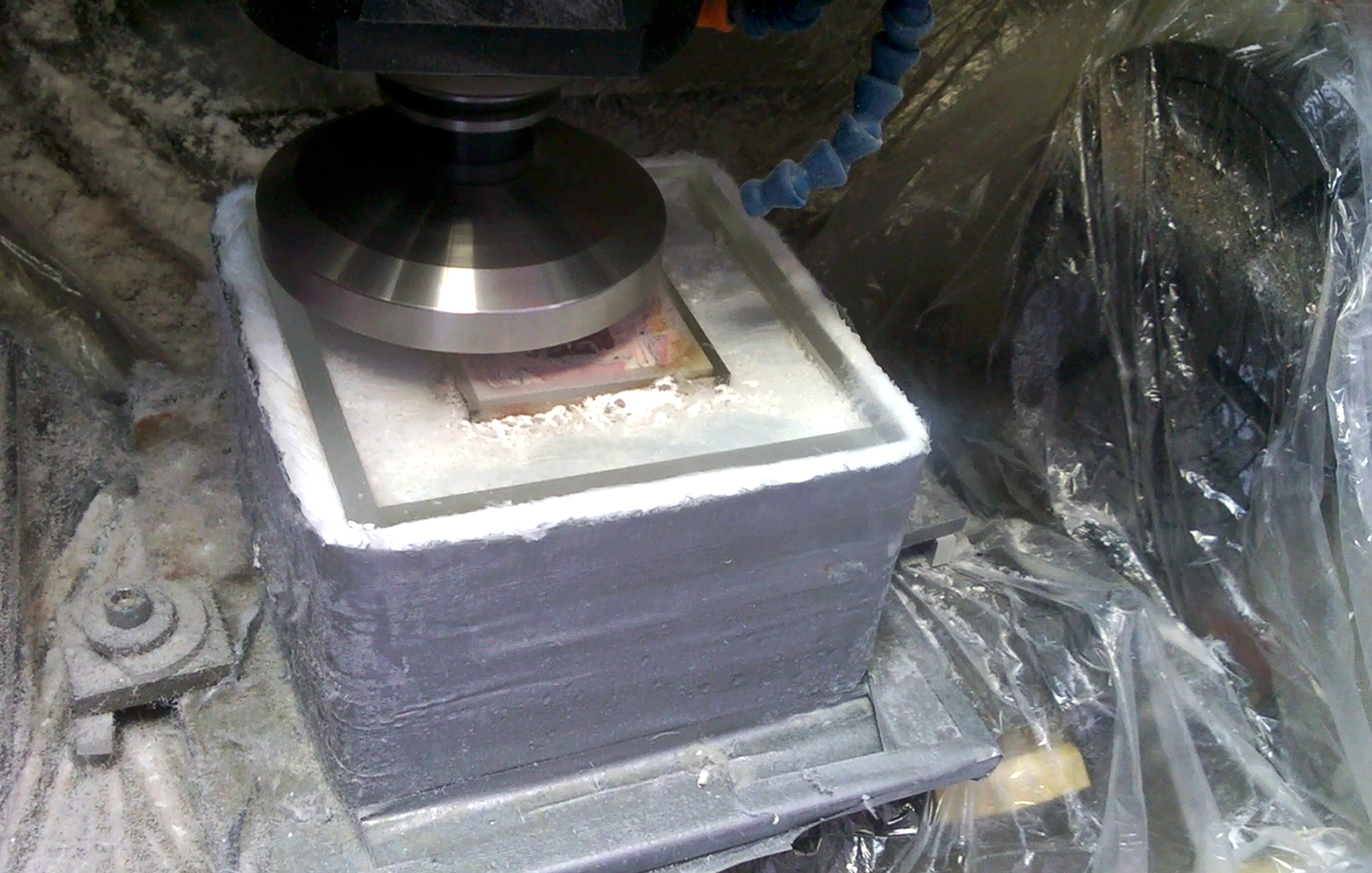

Supplement: S2 Fig — (TIF) [file pone.0213458.s002.tif]
